# The rhythm is going to get you

**DOI:** 10.7554/eLife.102428

**Published:** 2024-08-27

**Authors:** Mohini Sengupta

**Affiliations:** 1 Department of Biology, Saint Louis University Saint Louis United States

**Keywords:** locomotion, spinal cord, interneurons, rhythmogenesis, recurrent inhibition, reciprocal inhibition, motor control, Zebrafish

## Abstract

Slow and fast movements are controlled by distinct sets of spinal V2a neurons with matching properties and connections.

**Related research article** Agha MA, Kishore S, McLean DL. 2024. Cell-type-specific origins of locomotor rhythmicity at different speeds in larval zebrafish. *eLife* **13**:RP94349. doi: 10.7554/eLife.94349.

Walking, running and other rhythmic movements require muscles in the legs, arms and back to contract at the right time and in the correct sequence. The muscles involved are activated by motor neurons, which in turn are controlled by neurons within the spinal cord. These spinal neurons are organized into distinct populations, and understanding how these neurons function has been a central question in the field of motor control for many years. Interestingly, the organization and genetic make-up of these neurons are conserved across vertebrates (Goulding, 2009; Sengupta and Bagnall, 2023), making it possible to apply knowledge across species.

Rhythmic movements can be defined in terms of two basic features: speed (fast rhythms or slow rhythms), and pattern (that is, the type and sequence of muscles activated). For example, running involves fast rhythms and a certain pattern of powerful muscle activation, while walking involves slow rhythms and a different pattern of mild muscle activation. It has been shown that motor neurons, and the muscle fibers they contact, are divided into distinct subgroups that have unique cellular properties that complement either slow and mild or fast and powerful movements (Figure 1 left; Purves et al., 2001; McLean and Dougherty, 2015). However, it is unclear if spinal neurons also form such subgroups. Moreover, the mechanisms by which spinal neurons create and maintain slow and fast rhythms and different patterns are not fully understood.

**Figure 1.**
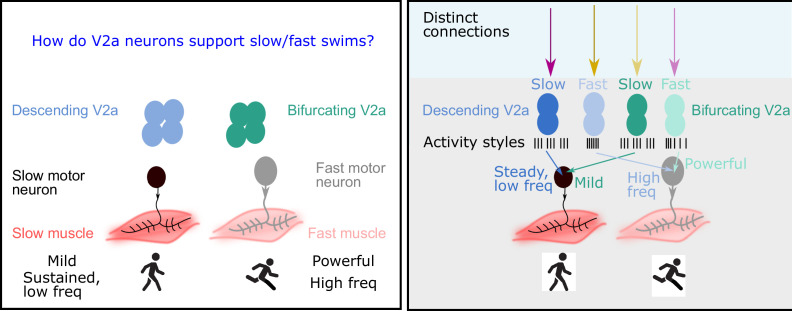
The role of V2a neurons in fast and slow movements. Left: The motor neurons that drive muscle movements receive inputs from V2a neurons in the central nervous system. These movements can be slow and steady (left) or fast and powerful (right). In 2019 it was shown that descending V2a neurons (blue) influence the speed of muscle movement, whereas bifurcating V2a neurons (green) influence the pattern of muscle activation (Menelaou and McLean, 2019). However, many details of these processes were not fully understood. Right: Now Agha et al. report that descending V2a neurons and bifurcating V2a neurons both come in two types: a slow type (dark blue or green) and a fast type (light blue or green). These different types also display different styles of electrical activity (black lines; each black line represents an action potential). The V2a neurons also receive inputs from excitatory and inhibitory neurons (to maintain their rhythm). The slow descending V2a neurons receive inputs from one source (magenta arrow), and their fast counterparts receive inputs from a different source (yellow arrow). Bifurcating neurons receive inputs from the opposite sources (lighter magenta and yellow arrows); moreover, these inputs are more broadly timed than those to the descending V2a neurons.

In 2019, two researchers at Northwestern University – Evdokia Menelaou and David McLean – discovered that spinal neurons called V2a neurons formed two distinct subpopulations: bifurcating V2a neurons (which have branched axons) influenced the pattern of muscle activation, while descending V2a neurons (which have a single axon) regulated the speed of the rhythm (Menelaou and McLean, 2019). Now, in eLife, Moneeza Agha, Sandeep Kishore and McLean report the results of experiments on zebrafish larvae that provide new insights into how V2a neurons implement slow or fast movements (Agha et al., 2024).

Previous studies in invertebrates have shown that the intrinsic activity of a neuron can be crucial for creating specific rhythms or patterns of movement (Marder and Bucher, 2001). Agha et al. studied zebrafish, a widely used vertebrate model organism, and employed genetic techniques to target V2a neurons specifically. Moreover, zebrafish larvae do not have a skull or any vertebrae, which makes it possible to record electrical signals directly from V2a neurons – a task that is extremely difficult to do in mice.

They found that descending V2a neurons come in two types: the first type displays continuous activity and is capable of generating fast rhythms; the second type displays bursts of activity, and maintains a slow but steady rhythm. Bifurcating V2a neurons also come in two types. Again, the first type generates fast rhythms, and the second generates bursts of slow rhythms.

Agha et al. then recorded the electrical activity of the V2a neurons as the zebrafish swam at different speeds, finding a remarkable relationship between the speed of movement and the activity of the different types of V2a neurons. For both descending and bifurcating V2a neurons, only the first type (the fast type) were active during the fast swims, while only the second type (the slow type) were active during the slow swims (Figure 1 right).

Next, Agha et al. explored how other neurons connected to the V2a neurons could affect their activity and ability to support different swim speeds by activating (exciting) or suppressing (inhibiting) the V2a neurons. For descending V2a neurons, the researchers found that both fast and slow types were contacted by excitatory and inhibitory partners. Unexpectedly, however, neurons of the first type (the fast type) were primarily inhibited by neurons that were located on the other side of the body (crossed inhibition), which helps them to maintain a fast rhythm, whereas neurons of the second type (slow) were inhibited by neurons on the same side of the body, which is preferable for maintaining a steady, slow rhythm (Figure 1 right).

In bifurcating V2a neurons, the opposite occurs: the fast type are inhibited by neurons on the same side of the body, while the slow type are inhibited by neurons on the opposite side. Moreover, there was more variability in the timing of the inhibitory/excitatory signals sent to the bifurcating V2a neurons, which is consistent with them being mostly involved in influencing the pattern of muscle activation, rather than controlling the speed of the rhythm (Figure 1 right).

Finally, Agha et al. studied zebrafish that had been genetically modified to remove an important source of crossed inhibition. As expected, this modification primarily impacted fast swims, proving that fast and slow movements do indeed require distinct types of V2a neurons (i.e., fast types and slow types) and their specific connecting partners.

These findings advance our knowledge in two key ways. First, they show that speed-specific groups of cells extend from the periphery (muscles) all the way into the central nervous system (spinal V2a neurons). Second, they provide a mechanistic understanding of how neurons combine their intrinsic activity and connections to execute different needs. So, you can walk or run, but you cannot hide, the spinal rhythms will get you.
